# A Variable Reduction Approach for Microbeams on Elastic Foundation

**DOI:** 10.3390/s25103034

**Published:** 2025-05-12

**Authors:** Giorgio Previati, Pietro Stabile, Federico Ballo

**Affiliations:** Department of Mechanical Engineering, Politecnico di Milano, Via La Masa 1, 20156 Milan, Italy; pietro.stabile@polimi.it (P.S.); federicomaria.ballo@polimi.it (F.B.)

**Keywords:** microbeam on elastic foundation, foundation gap modeling, nonlinear elastic foundation

## Abstract

In this paper, the bending behavior of microbeams resting on elastic foundations is analyzed. Due to the widespread use of Micro-Electro-Mechanical Systems (MEMSs) in sensing and actuation applications, various approaches have been developed for modeling such beams. Numerous specialized analytical and numerical models exist for specific configurations of beams and elastic foundations. This work proposes a novel approach. Separate models for the beam and the elastic foundation are developed using the finite element method. These models are then coupled using a variable reduction technique, in which only the degrees of freedom of the beam are retained in the solving system. This approach enables the coupling of any beam and foundation model and allows for independent refinement of the foundation mesh without increasing the size of the solving system. This method is particularly effective for analyzing configurations where the substrate exhibits nonlinear or non-homogeneous characteristics, or where gaps are present between the beam and the substrate. The nonlocal effects due to the small scale of the beam are also considered. This paper focuses on both the static deformation and frequency response of the microbeam. The proposed approach is validated against previously published models. Compared with existing models, the method presented here offers a simpler and more flexible formulation, while allowing the inclusion of nonlinearities in both the beam and foundation, as well as the modeling of gaps.

## 1. Introduction

Nano-microbeam mechanical systems are widely employed in the design and realization of Micro-Electro-Mechanical System (MEMS) devices. The general dimensions of MEMS devices typically range between tens and hundreds of microns and their working principle may involve different physical phenomena such as piezoelectricity, thermodynamics, and electromagnetism [1]. In general, the same principles can be used to enforce the motion of a micro-structure for designing micro actuators, or be exploited in the opposite way for sensing purposes [2,3,4,5,6,7,8,9].

An accurate model of the mechanical behavior of microbeams subject to transverse loading is crucial for the design of MEMS devices. However, below a certain scale, classical continuum mechanics theories are unable to model the structural behavior of such structures in a satisfactory way [10]. The common way to include these effects related to the small scale is that of reformulating classical beam equations according to a nonlocal elasticity theory, in which the stress at a given point in the material also depends on the strain acting at other points because of long-range interatomic interactions [11]. This yields to the modified constitutive beam equations that can be found, for instance, in [12,13,14,15,16].

Nonlocal beam models have been extensively applied for the solution of static and dynamic deflection of microbeams suspended over a fixed substrate, which constitute the basic structural configuration of MEMS devices [8,17,18,19]. In [8], the authors studied the dynamic response of a clamped-clamped microbeam to a mechanical shock. The equation of motion that governs the transverse displacement of the microbeam is solved by applying a Galerkin procedure. Their results show that the combination of shock loads with electrostatic actuation can activate low-frequency pull-in instability.

The same structural configuration was studied in [17], where the authors studied the effect of van der Waals forces on the pull-in instability voltage of electrostatically actuated micro- and nanobeams. In [18], different microbeam boundary conditions were also analyzed.

Sometimes the microbeam can come into contact with another substrate layer with different stiffness properties, such as the case of tactile sensors similar to the one presented in [20]. In these cases, nonlocal beam models can be coupled with (nonlinear) elastic foundations, which can be effectively employed to solve problems related to the adhesion mechanism in soft matter applications [21] or nonlinear contact problems [22,23].

Aside from the mentioned nonlocal beam theories based on classical continuum mechanics, an alternative approach for dealing with nonlocal material modelling is peridynamics, introduced by Silling in 2000 for modeling discontinuities in materials [24]. In peridynamics, partial differential equations are replaced by integro-differential equations to model long-range force/moment interactions [24,25,26], making the approach particularly effective in modeling discontinuities such as cracks (where partial derivatives are not defined). Although originally derived for modeling discontinuities in the material, peridynamics theory has also proved to be effective in modelling the structural behavior of micro-nanostructures. Examples of analytical and numerical solutions of peridynamic microbeams under bending can be found in [25,27,28], and in some cases, it is combined with classical theory, like in [26].

Regarding MEMS applications, an example of a microbeam resting on a foundation can be found in [29], where the vibration of an MEMS resonator past pull-in stitching is modeled by means of a clamped-clamped microbeam in contact with an elastic foundation describing the substrate. An accurate modeling of this particular condition can be useful for developing micro-switches and impact electrostatic actuators or developing stitching failure repair vibration strategies.

In [10,30], a general formulation of the boundary value problem of a micro- or nanobeam vibrating on an elastic foundation is presented. The structural effect of the small scale of the nanobeam is included in the Euler-Bernoulli formulation by applying Eringen’s nonlocal elasticity theory. The nonlinear effect due to stretching of the neutral axis of the beam is included as well.

Similar equations were derived by Demir et al. in [14,31] and by Kacar et al. [32], where the static and dynamic deflection of a nanobeam resting on a Winkler foundation was computed. In the papers, the authors leveraged either the differential transform [14,32] or the Galerkin weighted residual [31] method to derive the finite element equations of the boundary value problem. For a constant stiffness foundation modulus, an explicit expression of the element stiffness matrix was obtained. Aside from the classical Euler-Bernoulli element stiffness matrix, two additional terms appeared, pertaining to the Winkler foundation and the nonlocal elasticity, respectively [31]. The Galerkin weighted residual method was also applied in [33,34], where the problem of vibration of a nanobeam resting on a three-layer nonlinear elastic foundation was solved.

In this paper, a novel formulation approach for the solution of microbeams resting on nonlinear elastic foundations is proposed. Starting from the nonlinear differential equation of the microbeam on the foundation, a Galerkin weighted residual method is applied to derive the corresponding system of equations at the discretized level. The element stiffness matrices and the vector of nodal forces are separately derived for the microbeam and for the foundation element.

The connection between the two element types is enforced by applying a variable reduction method, in which the Jacobian matrix that relates the nodal degrees of freedom of the beam to those of the foundation is explicitly derived from the shape functions employed in the beam element.

The main advantage of the proposed method lies in the possibility to completely decouple the contributions of the beam and the elastic foundation, enabling the use of completely independent meshes for the beam and the foundation in the discretized problem. This feature could be significantly convenient in modeling sandwich-type non-homogeneous microbeams, such as the ones in [35], in piezoelectrically actuated devices [2], or in modeling stitching and post-stitching deformations in [29]. Moreover, the method is quite general and applies to any combination of beam and foundation elements, and may also include gap-induced nonlinearities in cases where the beam loses contact with the substrate.

The structure of the paper is as follows. The general analytical formulation of a microbeam resting on an elastic foundation and subject to a transverse load is described in Section 2. Both the equations referring to the static displacement and the dynamic vibration of the beam are recalled. In Section 3, the continuous differential equation is integrated by means of the Finite Element Method (FEM). The expressions of the element stiffness matrix and equivalent nodal load vectors are separately derived for the beam and for the elastic foundation. In Section 4, the assembly procedure of the resulting system is described, in which only the nodal degrees of freedom of the beam are retained thanks to the use of a variable reduction approach. In Section 5, two different numerical examples are presented to validate the proposed method. The first example refers to the computation of the natural frequencies of a hinged-hinged microbeam resting on an elastic foundation; different test cases, including both linear and nonlinear elastic foundations, are solved. The second example involves the computation of the dynamic vibration of a clamped-clamped microbeam on a nonlinear elastic foundation with an initial gap, a typical problem encountered in modeling MEMS devices. Finally, conclusions are drawn in Section 6.

## 2. Analytical Formulation

**Figure 1:**
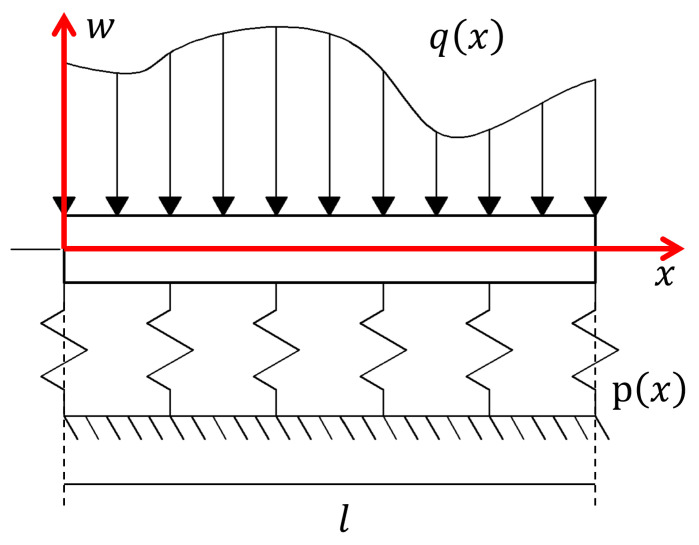
Microbeam on an elastic foundation. The schematic representation of the microbeam resembles the representation of a standard beam. Please note that the actual dimensions of the microbeam are in the microscale range (fractions of a millimeter).

A general microbeam resting on a given elastic foundation is depicted in Figure 1. An arbitrary distributed load $q\left(x\right)$, which is a function of the axial coordinate *x*, is applied to the beam of length *l*. Assuming a general foundation characterized by a function $p\left(x\right)$, and according to the Euler-Bernoulli beam theory, the vertical displacement of the beam $w\left(x\right)$ can be related to the internal moment as [13,31].

(1) $$EI\frac{d^{2}w\left(x\right)}{dx^{2}}+M\left(x\right)-{\left(e_{0}a\right)}^{2}\frac{d^{2}M\left(x\right)}{dx^{2}}=0\;\forall x\in \left[0,l\right],$$

with

(2) $$\frac{d^{2}M\left(x\right)}{dx^{2}}=-q\left(x\right)+p\left(x\right),$$

where ${\left(e_{0}a\right)}^{2}$ is the nonlocal parameter, *E* the elastic modulus of the material, and *I* the moment of inertia of the cross-section. The second derivative of the bending moment in Equation (2) results from the analysis of the stress resultants in nonlocal theories, as explained in [12,13,36].

By substituting Equation (2) into Equation (1), the expression of the internal moment reads

(3) $$M\left(x\right)=-EI\frac{d^{2}w\left(x\right)}{dx^{2}}+{\left(e_{0}a\right)}^{2}\left(-q\left(x\right)+p\left(x\right)\right).$$

Finally, by substituting Equation (3) into Equation (2), the Euler-Bernoulli formulation of a microbeam subjected to an arbitrary distributed load resting on a general elastic foundation can be obtained as [13,31].

(4) $$EI\frac{d^{4}w\left(x\right)}{dx^{4}}+\left({\left(e_{0}a\right)}^{2}\frac{d^{2}q\left(x\right)}{dx^{2}}-q\left(x\right)\right)-\left({\left(e_{0}a\right)}^{2}\frac{d^{2}p\left(x\right)}{dx^{2}}-p\left(x\right)\right)=0\;\forall x\in \left[0,l\right],$$

Equation (4) has three terms, namely:

- $EI\frac{d^{4}w\left(x\right)}{dx^{4}}$: This term is related to the structural stiffness of the beam and has the classical expression for the Euler-Bernoulli formulation. If shear is also considered in the beam formulation, an additional term is included, and the Timoshenko beam formulation can be obtained [15].
- ${\left(e_{0}a\right)}^{2}\frac{d^{2}q\left(x\right)}{dx^{2}}-q\left(x\right)$: This term describes the contribution of the applied distributed load. If the distributed load has non-vanishing derivatives, a nonlocal effect is present in the differential formulation.
- ${\left(e_{0}a\right)}^{2}\frac{d^{2}p\left(x\right)}{dx^{2}}-p\left(x\right)$: This term is related to the elastic foundation. Also, for the elastic foundation, a nonlocal term is present [13].

Equation (4) is general and can describe any elastic foundation formulation. Only in very few cases can this expression be analytically integrated, leading to specialized formulations.

Equation (4) refers to a static analysis. By considering the material density ρ and the cross-sectional area *A*, the vibration of the structure for a given small displacement $w_{0}$ can be computed as

(5) $$\begin{array}{c} \;EI\frac{d^{4}w\left(x\right)}{dx^{4}}-\left({\left(e_{0}a\right)}^{2}\frac{d^{2}p\left(x\right)}{dx^{2}}-p\left(x\right)\right)=\rho A\omega^{2}w-{\left(e_{0}a\right)}^{2}\rho A\omega^{2}\frac{d^{2}w}{dx^{2}}\\ \;\forall x\in \left[0,l\right], \end{array}$$

where ω is the natural frequency of vibration (for details on the vibrations of microbeams and on how to derive the differential expression, see [12]). The term $\rho A\omega^{2}w$ is the usual mass contribution to the vibration of a continuous beam, while the term $-{\left(e_{0}a\right)}^{2}\rho A\omega^{2}\frac{d^{2}w}{dx^{2}}$ represents the nonlocal contribution.

In the following, a simple and general numerical approach will be described for the finite element integration of the differential equations.

## 3. Finite Element Numerical Solution

The numerical integration of Equation (4) is performed using the finite element method (FEM). For this application, two different meshes are considered: one to discretize the beam and another for the discretization of the elastic foundation. As discussed in [37], when analyzing a beam on an elastic foundation, the stiffness matrix of the beam is modified by incorporating the contribution of the elastic foundation. However, if the two contributions are treated separately, a stiffness matrix can be defined for the beam and another for the foundation. This approach allows for the definition of an element that represents the foundation’s characteristics, which can then be assembled into the system matrix. In this work, the contributions are treated separately using distinct elements for the beam and the foundation. This separation enables the independent definition of the two meshes, allowing for different discretizations to be applied to the beam and the elastic foundation.

### 3.1. Finite Element Model of the Beam

For the FEM analysis of the beam, the standard two-node beam element with Hermite cubic polynomials is used. Each node has two degrees of freedom (DOFs): the transverse displacement $w_{i}$ and the rotation $\theta_{i}$, where i=1,2 corresponds to the first node (i=1) and the second node (i=2). Therefore, the vector of nodal displacements is

(6) $$d={\left[\begin{array}{cccc} w_{1} & \theta_{1} & w_{2} & \theta_{2} \end{array}\right]}^{T}$$

and the four Hermite cubic polynomial shape functions $N\left(x\right)$ of the element are given by

(7) $$N\left(x\right)=\left[\begin{array}{cccc} \frac{2x^{3}}{L^{3}}-\frac{3x^{2}}{L^{2}}+1, & x-\frac{2x^{2}}{L}+\frac{x^{3}}{L^{2}}, & \frac{3x^{2}}{L^{2}}-\frac{2x^{3}}{L^{3}}, & \frac{x^{3}}{L^{2}}-\frac{x^{2}}{L} \end{array}\right]$$

where *x* is the axial coordinate of the element, and *L* is its length.

The numerical integration of the structural stiffness term in Equation (4) leads to the well-known expression for the stiffness matrix of the Euler-Bernoulli beam, which is given by

(8) $$K_{B}=\frac{EI}{L}\left[\begin{array}{cccc} \frac{12}{L^{2}} & \frac{6}{L} & -\frac{12}{L^{2}} & \frac{6}{L}\\ \frac{6}{L} & 4 & -\frac{6}{L} & 2\\ -\frac{12}{L^{2}} & -\frac{6}{L} & \frac{12}{L^{2}} & -\frac{6}{L}\\ \frac{6}{L} & 2 & -\frac{6}{L} & 4 \end{array}\right].$$

For the computation of the load term $F_{q}$ due to the contribution of the distributed load, including the nonlocal effect, the weak Galerkin approach can be used. If an arbitrary weight function $W\left(x\right)$ is considered, the weak formulation of the distributed load contribution is given by

(9) $$\begin{array}{c} F_{q}={\left(e_{0}a\right)}^{2}\int_{0}^{L}W\left(x\right)\frac{d^{2}q\left(x\right)}{dx^{2}}dx+\int_{0}^{L}W\left(x\right)q\left(x\right)dx=\\ {\left(e_{0}a\right)}^{2}\int_{0}^{L}\frac{d^{2}W\left(x\right)}{dx^{2}}q\left(x\right)dx+\int_{0}^{L}W\left(x\right)q\left(x\right)dx \end{array}$$

By considering the standard FEM approach, the transverse displacement and the test function can be defined as

(10) $$w\left(x\right)=N\left(x\right)d\;\;\;\;\;\;W\left(x\right)=N\left(x\right)$$

By replacing Equation (10) in Equation (9), $F_{q}$ can be computed as

(11) $$F_{q}={\left(e_{0}a\right)}^{2}\int_{0}^{L}\frac{d^{2}N\left(x\right)}{dx^{2}}q\left(x\right)dx+\int_{0}^{L}N\left(x\right)q\left(x\right)dx$$

If the distributed load is constant, i.e., $q\left(x\right)=q_{0}$, $F_{q}$ can be analytically computed and reads [31]

(12) $$F_{q}={\left(e_{0}a\right)}^{2}q_{0}\left[\begin{array}{c} 0\\ -1\\ 0\\ 1 \end{array}\right]+q_{0}\left[\begin{array}{c} \frac{L}{2}\\ \frac{L^{2}}{12}\\ \frac{L}{2}\\ -\frac{L^{2}}{12} \end{array}\right]$$

On the other hand, if the distributed load is not constant with respect to *x*, Equation (11) generally cannot be analytically integrated. The integration of Equation (11) must be performed numerically using the Gauss quadrature method, and $F_{q}$ can be computed as

(13) $$F_{q}={\left(e_{0}a\right)}^{2}\sum_{i=1}^{n}\omega_{i}q\left(x_{i}\right){\left.\frac{d^{2}N\left(x\right)}{dx^{2}}\right|}_{x=x_{i}}+\sum_{i=1}^{n}\omega_{i}q\left(x_{i}\right)N\left(x_{i}\right)$$

where *n* is the number of Gauss points in the element, $\omega_{i}$ the integration weights, and $x_{i}$ the Gauss point locations.

The two terms representing the mass contributions in Equation (5) can be computed as follows. The first term is the well-known mass term associated with the Euler-Bernoulli beam model and reads

(14) $$M_{B}=\frac{\rho AL}{420}\left[\begin{array}{cccc} 156 & 22L & 54 & -13\\ 22L & 4L^{2} & 13L & -3L^{2}\\ 54 & 4L^{2} & 36 & -22L\\ 4L^{2} & -3L^{2} & -22L & 4L^{2} \end{array}\right].$$

The second term of the mass contribution can be derived by the Galerkin method as

(15) $$M_{S}=-{\left(e_{0}a\right)}^{2}\int_{0}^{L}N^{T}\left(x\right)\rho A\frac{d^{2}N\left(x\right)}{dx^{2}}dx={\left(e_{0}a\right)}^{2}\rho A\int_{0}^{L}\frac{d^{T}N\left(x\right)}{dx}\frac{dN\left(x\right)}{dx}dx$$

Equation (15) can be integrated analytically as

(16) $$M_{S}={\left(e_{0}a\right)}^{2}\frac{\rho A}{30L}\left[\begin{array}{cccc} 36 & 3L & -36 & 3L\\ 3L & 4L^{2} & -3L & -L^{2}\\ -36 & -3L & 36 & -3L\\ 3L & -L^{2} & -3L & 4L^{2} \end{array}\right].$$

Finally, the mass matrix results:

(17) $$M_{S}=M_{B}+M_{S}.$$

It is worth noting that the proposed numerical integration was presented for a specific choice of element DOFs and shape function expressions. The same formal procedure can be applied to any other element formulation, i.e., DOF definitions and shape function expressions, provided that it satisfies the requirements of the FEM.

### 3.2. Finite Element Model of the Elastic Foundation

For the finite element modeling of the elastic foundation elements, a two-node beam element, of length $L_{f}$, with third-order Hermite shape functions is considered. By this choice, the same element is used both for the beam and for the foundation elements. However, the same formal procedure can be applied to any other kind of element complying with the finite element requirements.

For the elastic foundation, the following nonlinear expression is considered:

(18) $$p\left(x\right)=k\left(x\right)w\left(x\right)$$

where $k\left(x\right)$ is the nonlinear stiffness coefficient. This expression corresponds to a nonlinear Winkler [16,38] foundation. According to [16], to consider the nonlocal effect of the nonlinear Winkler foundation, the second derivative of Equation (18) has to be considered, and it reads

(19) $$\frac{d^{2}\left(k\left(x\right)w\left(x\right)\right)}{dx^{2}}=k\left(x\right)\frac{d^{2}w\left(x\right)}{dx^{2}}+2\frac{dk\left(x\right)}{dx}\frac{dw\left(x\right)}{dx}+\frac{d^{2}k\left(x\right)}{dx^{2}}w\left(x\right)$$

By considering the expression of the elastic foundation of Equation (18) and its derivative of Equation (19), the term $R_{p}$ due to the contribution of the elastic foundation of Equation (4) can be obtained by the Galerkin method and it reads

(20) $$\begin{array}{c} R_{p}=\left(\int_{0}^{L_{f}}k\left(x\right)N^{T}\left(x\right)N\left(x\right)dx+{\left(e_{0}a\right)}^{2}\int_{0}^{L_{f}}k\left(x\right)\frac{N^{T}\left(x\right)}{d}\frac{dN\left(x\right)}{d}dx+\right.\;\\ +\left.{\left(e_{0}a\right)}^{2}\int_{0}^{L_{f}}\frac{dk\left(x\right)}{dx}\frac{dN^{T}\left(x\right)}{dx}N\left(x\right)dx\right)d_{f} \end{array}$$

where $d_{f}$ are the nodal displacements of the foundation element and, for each foundation element, $w\left(x\right)=N\left(x\right)d_{f}$.

$R_{p}$ has three terms. The first term is the usual stiffness contribution for the local model of a beam on an elastic foundation. The second two terms are derived from the nonlocal model of the beam. Interestingly, the third term contains the derivatives of the stiffness of the elastic foundation. This term is present only if a nonlinear foundation is considered.

By defining the foundation stiffness matrix $K_{p}\left(x\right)$, $R_{p}$ can be expressed in compact form as

(21) $$R_{p}=K_{p}\left(x\right)d_{f}$$

If $k\left(x\right)$ is constant and equal to $k_{0}$, $K_{p}\left(x\right)$ is constant as well, and it reads [31]

(22) $$\begin{array}{c} K_{P}=\frac{k}{420}\left[\begin{array}{cccc} 156L & 22L^{2} & 54L & -13L^{2}\\ 22L^{2} & 4L^{3} & 13L^{2} & -3L^{3}\\ 54L & 13L^{2} & 156L & -22L^{2}\\ -13L^{2} & -3L^{3} & -22L^{2} & 4L^{3} \end{array}\right]+\\ \;+\frac{{\left(e_{0}a\right)}^{2}k}{30L}\left[\begin{array}{cccc} 36 & 3L & -36 & 3L\\ 3L & 4L^{2} & -3L & -L^{2}\\ -36 & -3L & 36 & -3L\\ 3L & -L^{2} & -3L & 4L^{2} \end{array}\right] \end{array}$$

If $k\left(x\right)$ is not constant, Gaussian integration can be used to compute $K_{p}\left(x\right)$ as

(23) $$\begin{array}{c} K_{p}\left(x\right)=\sum_{i=1}^{n}\omega_{i}k\left(x_{i}\right)N^{T}\left(x_{i}\right)N\left(x_{i}\right)+\\ \;+{\left(e_{0}a\right)}^{2}\sum_{i=1}^{n}\omega_{i}k\left(x_{i}\right){\left.\frac{dN^{T}\left(x\right)}{dx}\right|}_{x=x_{i}}{\left.\frac{dN\left(x\right)}{dx}\right|}_{x=x_{i}}+\\ \;+{\left(e_{0}a\right)}^{2}\sum_{i=1}^{n}\omega_{i}{\left.\frac{dk\left(x\right)}{dx}\right|}_{x=x_{i}}{\left.\frac{dN^{T}\left(x\right)}{dx}\right|}_{x=x_{i}}N\left(x_{i}\right) \end{array}$$

### 3.3. Finite Element Model of the Gap

In case an initial gap $w_{0}\left(x\right)$ is present between the beam and the foundation, Equation (18) can be modified as

(24) $$p\left(x\right)=k\left(x\right)\left(w\left(x\right)-w_{0}\left(x\right)\right)$$

where for a unilateral contact, the stiffness coefficient can be described by

(25) $$k\left(x\right)=\left\{\begin{array}{c} 0\;if\;\;w\left(x\right)-w_{0}\left(x\right)>0\\ k\left(x\right)\;if\;\;w\left(x\right)-w_{0}\left(x\right)\leq 0 \end{array}\right.\;\forall x\in \left[0,L_{f}\right],$$

Equation (25) states that the elastic foundation stiffness is considered only when the beam is actually in contact with the foundation, and separation is allowed between the beam and the foundation.

It must be emphasized that the contact area is, in general, not known a priori and must be computed during the solution of the system. Since the contact area depends on the displacements, the system is inherently nonlinear [39]. Equation (25) must be checked at each iteration of the solution and at each computation point to determine the actual contact area.

The initial gap can be expressed as

(26) $$w_{0}\left(x\right)=N\left(x\right)d_{0,f}$$

with $d_{0,f}$ being the vector of the gap values at the nodes of the foundation element. By defining the quantity $\hat{w}\left(x\right)=w\left(x\right)-w_{0}\left(x\right)=N\left(x\right)\left(df-d0,f\right)$, Equation (19) can be rewritten as

(27) $$\frac{d^{2}\left(k\left(x\right)\hat{w}\left(x\right)\right)}{dx^{2}}=k\left(x\right)\frac{d^{2}\hat{w}\left(x\right)}{dx^{2}}+2\frac{dk\left(x\right)}{dx}\frac{d\hat{w}\left(x\right)}{dx}+\frac{d^{2}k\left(x\right)}{dx^{2}}\hat{w}\left(x\right)$$

which is expressed in Galerkin form as

(28) $$\begin{array}{c} R_{pg}=\left(\int_{0}^{L_{f}}k\left(x\right)N^{T}\left(x\right)N\left(x\right)dx+{\left(e_{0}a\right)}^{2}\int_{0}^{L_{f}}k\left(x\right)\frac{N^{T}\left(x\right)}{d}\frac{dN\left(x\right)}{d}dx+\right.\\ +\left.{\left(e_{0}a\right)}^{2}\int_{0}^{L_{f}}\frac{dk\left(x\right)}{dx}\frac{dN^{T}\left(x\right)}{dx}N\left(x\right)dx\right)\left(d_{f}-d_{0,f}\right) \end{array}$$

In compact form, Equation (28) can be written as

(29) $$R_{pg}=K_{p}\left(x\right)d_{f}-K_{p}\left(x\right)d_{0,f}$$

with $K_{p}$ being the stiffness matrix of the foundation element.

## 4. Solution of the Finite Element Model by a Variable Reduction Approach

In the previous sections, the finite element model of a microbeam resting on a nonlinear elastic foundation with a gap was derived, considering different meshes for the beam and the foundation. Using distinct meshes allows for accurate modeling of both the beam and the foundation, enabling the application of the most appropriate and effective discretization and element type for each. For the solution of the finite element system, the two meshes have to be assembled, and the solving system has to be constructed. In the literature, the most common approach for assembling the beam and foundation is to use the same nodes for the two meshes, i.e., the same mesh for the two parts. In [40], different meshes are used for the two parts and then assembled by constructing a series of constraint conditions by using Lagrange multipliers. This approach allows for using different discretizations and different element types. However, the use of Lagrange multipliers results in a mixed formulation and a more complex numerical solution. In the following, the variable reduction approach defined in [23] for beams resting on an elastic foundation is extended to the case of microbeams with a nonlocal effect.

In the variable reduction approach, kinematic constraints are enforced on the DOFs of the foundation element nodes, leaving only the DOFs of the beam element nodes as free DOFs considered in the model’s solving system. The variable reduction approach is well established in the finite element method, and the resulting system can be solved by the usual techniques for the solution of nonlinear systems.

**Figure 2:**
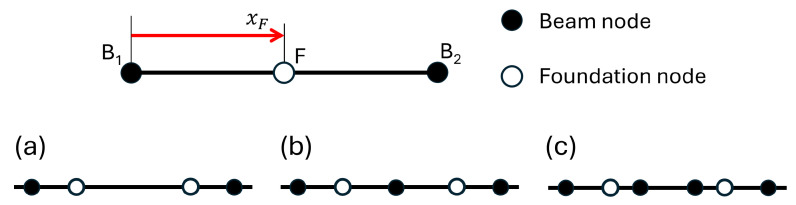
Definition of the relative position of the elastic foundation node with respect to the beam’s nodes. Top: position of an elastic foundation node F on the axis of a beam element with nodes B_1_ and B_2_, where $x_{F}$ is the position of the node F on the beam axis in the beam’s reference system. Bottom: possible relative position of an elastic foundation element with respect to the beam elements.

The displacements of an elastic foundation node F constrained to lie on the axis of a beam element of nodes B_1_ and B_2_, depicted at the top of Figure 2, can be computed as

(30) $$\left\{\begin{array}{c} w_{F}=N\left(x_{F}\right)d_{\mathrm{beam}}\\ \theta_{F}=N^{I}\left(x_{F}\right)d_{\mathrm{beam}} \end{array}\right.\Rightarrow d_{F}=\Lambda_{F}\left(x_{F}\right)d_{\mathrm{beam}}$$

where $d_{\mathrm{beam}}={\left[w_{1}\;\;\theta_{1}\;\;w_{2}\;\;\theta_{2}\right]}^{T}$ indicates the vector of the nodal displacements of the beam element, with $w_{1}$ and $\theta_{1}$ and $w_{2}$ and $\theta_{2}$ denoting the displacements and rotations of the nodes B_1_ and B_2_, respectively, and $d_{F}={\left[w_{F}\;\;\theta_{F}\right]}^{T}$ the displacement and rotation of the node F of the elastic foundation. $\Lambda_{F}\left(x_{F}\right)=\left[\begin{array}{c} N\left(x_{F}\right)\\ N^{I}\left(x_{F}\right) \end{array}\right]$ is the Jacobian matrix relating the DOFs of node F and the displacements of the nodes of the beam element, and $x_{F}$ is the coordinate of the node F on the beam element.

At the bottom of Figure 2, the three possible relative positions of the nodes of a foundation element with respect to the beam mesh are depicted. In Case (a), both nodes of the foundation element belong to the same beam element, resulting in identical deformations if the same shape functions are used, with no discretization error. This is the optimal case, especially when the foundation and beam nodes coincide, requiring the foundation mesh to include all beam nodes. In Case (b), one foundation node belongs to one beam element and the other to a neighboring element, causing slight deformation differences that decrease as foundation elements are made smaller. This case is acceptable, and convergence analysis can optimize mesh dimensions. In Case (c), one node belongs to a non-neighboring beam element, leading to mismatched deformations and uncontrollable errors, making it unacceptable. To minimize errors, foundation meshes should have smaller or equal-sized elements compared to beam meshes and contain more or equal numbers of elements.

On the basis of Equation (30), the relationship between the displacements $d_{1}$ and d2 of the nodes of a foundation element and the displacements dbeam of the nodes of the beam mesh can be expressed as

(31) $$\left\{\begin{array}{c} d_{1}=\Lambda_{d1}\left(x_{d_{1}}\right)d_{\mathrm{beam}}\\ d_{2}=\Lambda_{d2}\left(x_{d_{2}}\right)d_{\mathrm{beam}} \end{array}\right.\Rightarrow d=\Lambda d_{\mathrm{beam}}$$

where $d=\left[\begin{array}{c} d_{1}\\ d_{2} \end{array}\right]$. Matrix Λ represents the global Jacobian matrix that relates the displacements of the two nodes of the foundation element to the displacements of the beam nodes. The matrix Λ is assembled from the two Jacobian matrices, Λd1 and Λd2, which are defined in Equation (30). These matrices describe the relationship between the displacements of each node of the foundation element and the displacements of the nodes of the corresponding beam element. To construct Λ, the matrices Λd1 and Λd2 are assembled using the standard assembly rules of the finite element method. This definition of Λ is applicable to any scenario illustrated at the bottom of Figure 2.

In Equation (29), the constitutive equations of the foundation element with a gap for a microbeam are expressed as a function of the displacements of the foundation element nodes. To apply the variable reduction method and obtain the constitutive equation as a function of the displacement of the beam mesh nodes, the following energy equivalence can be considered.

(32) $$\delta d_{beam}^{T}R_{pg,beam}=\delta d_{f}^{T}R_{pg}$$

where $\delta d_{beam}$ and $\delta d_{f}$ are arbitrary virtual displacements of the beam mesh nodes and of the foundation element nodes, respectively, $R_{pg,beam}$ indicates the foundation element constitutive equations as function of the beam mesh nodes, and $R_{pg}$ is given in Equation (29).

By considering Equation (29), Equation (32) can be rewritten as

(33) $$\delta d_{beam}^{T}R_{pg,beam}=\delta d_{f}^{T}K_{p}\left(x\right)d_{f}-\delta d_{f}^{T}K_{p}\left(x\right)d_{0,f}$$

By replacing Equation (31) into Equation (33), the following relationship can be obtained:

(34) $$\begin{array}{c} \delta d_{beam}^{T}R_{pg,beam}=\delta d_{\mathrm{beam}}^{T}\Lambda^{T}K_{p}\left(x\right)\Lambda d_{\mathrm{beam}}-\delta d_{\mathrm{beam}}^{T}\Lambda^{T}K_{p}\left(x\right)\Lambda d_{0,\mathrm{beam}} \end{array}$$

where the initial gap at the nodes of the foundation elements is expressed as $d_{0,f}=\Lambda d_{0,\mathrm{beam}}$, with $d_{0,\mathrm{beam}}$ being the initial gap at the nodes of the beam elements.

Finally, by simplifying the arbitrary virtual displacement $\delta d_{beam}^{T}$ in Equation (34), the expression of the constitutive equations of the foundation element with a gap for a microbeam as a function of the displacements of the beam nodes can be written as

(35) $$R_{pg,beam}=K_{p,beam}\left(x\right)d_{beam}-K_{p,beam}\left(x\right)d_{0,beam}$$

where

(36) $$K_{p,\mathrm{beam}}\left(x\right)=\Lambda^{T}K_{p}\left(d_{f}\right)\Lambda$$

Equation (36) can be computed from Equation (23) by remembering $d_{f}=\Lambda d_{\mathrm{beam}}$ (Equation (31)).

Equation (35) represents the constitutive equation of the foundation element as a function of only the DOFs of the beam mesh. This equation demonstrates that the solving system depends solely on the beam’s DOFs and, therefore, its dimensionality remains unchanged as the foundation mesh is refined. From a computational perspective, the time required to solve the system is independent of the size of the foundation mesh. However, there is an increase in the time needed to assemble the solving system, as Equation (35) must be computed for each element of the foundation mesh. The increase in computational time for the assembly process is linear with the number of foundation elements. The nodal displacements of the foundation mesh elements can be computed from the solution of the displacements of the beam mesh in a post-processing phase by applying Equation (31).

In general, the terms of Equation (35) are a function of the nodal displacements and the resulting system is nonlinear. In particular, the system is always nonlinear if a gap is present. The solution of the nonlinear system is outside the scope of this paper. As the approach used in this paper complies with the usual finite element requirements, any solution technique for solving nonlinear finite element systems available in the literature can be applied. In the following, a simple fixed-point iteration method [41] is used.

## 5. Numerical Examples

In this section, two numerical examples are presented to show the potential of the presented model. The first example is taken from the literature and used to validate the elastic foundation model. This particular example does not benefit from the possibility of using different meshes due to uniform properties of the foundation and no gap. The second example is a beam on an elastic foundation with a gap. In this case, a selective refinement of just the foundation mesh can be applied to improve accuracy.

### 5.1. Example 1: Foundation Model Validation

This example is used to validate the nonlinear elastic foundation model and it is taken from the literature. The example involves the computation of the natural frequencies of the hinged-hinged microbeam on a nonlinear elastic foundation discussed in [16]. In [16], the problem is solved by using the Differential Quadrature Method developed in [42]. In the paper, the cases of graded microbeams and clamped-hinged microbeams are also considered. For the sake of space, in this example, only uniform hinged-hinged microbeams are considered. As the geometry is quite simple and both the microbeam and elastic foundation have uniform properties along the axis of the beam, the same mesh is used for both. In this case, the nodes of the foundation mesh coincide with the nodes of the beam mesh, and the Jacobian matrix in Equation (31) reduces to the identity matrix.

In [16], the following four expressions for the foundation stiffness $k\left(x\right)$ are considered.

- Constant stiffness: $k\left(x\right)=k_{0}$
- Linear stiffness: $k\left(x\right)=k_{0}\left(1-\alpha \frac{x}{L}\right)$
- Quadratic stiffness: $k\left(x\right)=k_{0}\left(1-\beta \frac{x^{2}}{L^{2}}\right)$
- Sinusoidal stiffness: $k\left(x\right)=k_{0}\left(1-\delta \sin\left(\frac{x}{L}\right)\right)$

Table 1 shows the comparison of the frequency parameter $\sqrt{\lambda }$ computed by the presented model and the reference model of [16] for a microbeam resting on a constant, linear, quadratic, and sinusoidal elastic foundation. For all of the considered cases, the two models provide the same results. The computations have been performed with E=1, ρ=1, I=1, A=1, ${\left(e_{0}a\right)}^{2}=0.5$, l=1, $k_{0}=500$, and α=β=δ=1. In Appendix A, additional comparisons are provided by varying the value of the nonlocal coefficient. A discussion of the effect of the nonlocal coefficient on the deformation of microbeams can be found in [43].

**Figure 3:**
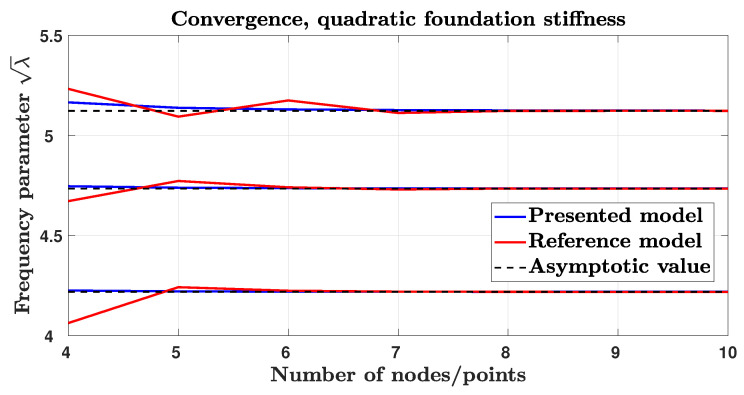
Convergence rate of the method presented in this paper is compared with the convergence rate of the method described in [16]. The case of a quadratic elastic foundation is considered.

In Figure 3, the convergence of the method presented in this paper is presented and compared with the method described in [16] for the case of the quadratic elastic foundation. The two methods show a similar convergence rate and both converge to the same value.

It is important to note that, although the method presented in [16] and the method presented in this paper show similar convergence performance and produce the same frequency parameter values, their flexibility differs significantly. In fact, if a different system of constraints needs to be analyzed, the Differential Quadrature Method necessitates significant modifications to the system matrices. In contrast, the method proposed in this paper, which is based on a standard finite element approach, only requires the removal of the pertinent rows and columns from the system matrices.

### 5.2. Example 2: Foundation with Gap

**Figure 4:**
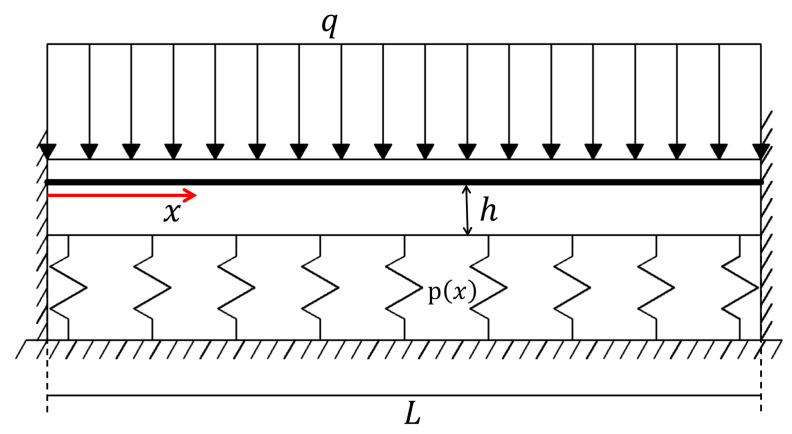
Clamped-clamped microbeam on nonlinear elastic foundation with gap.

In this section, a clamped-clamped microbeam on a nonlinear elastic foundation with a gap is considered. The nonlinear foundation has a sinusoidal stiffness described by the expression $k\left(x\right)=k_{0}\left(1-\delta \sin\left(\frac{\pi x}{L}\right)\right)$. The scheme of the beam is shown in Figure 4, and the considered data are E=150000MPa, $\rho =2.5\cdot 10^{-6}\;\frac{\mathrm{kg}}{{\mathrm{mm}}^{3}}$, $I=1.707\cdot 10^{-11}\;{\mathrm{mm}}^{4}$, $A=8\cdot 10^{-5}\;{\mathrm{mm}}^{2}$, L=0.25mm, ${\left(e_{0}a\right)}^{2}=0.5$, $k_{0}=32.768\;\frac{N}{{\mathrm{mm}}^{2}}$, and δ=0.5. A constant distributed load $q=5\cdot 10^{-3}\;\frac{N}{\mathrm{mm}}$ is also applied to the beam. The gap distance is $h=0.5\cdot 10^{-3}\;\mathrm{mm}$.

**Figure 5:**
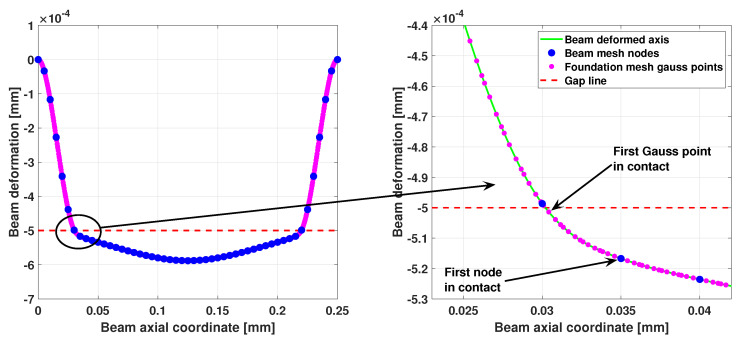
Deformed shape of the microbeam on nonlinear foundation with gap. (**Left**): complete deformation. (**Right**): detail of the left initial contact zone.

**Figure 6:**
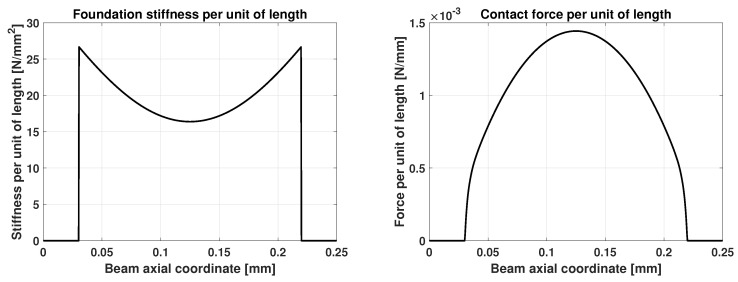
(**Left**): foundation stiffness per unit of length. (**Right**): foundation contact force per unit of length.

Figure 5 shows the deformation of the microbeam, computed using 50 beam elements and 200 foundation elements, with four Gauss points each used for the numerical integration of the foundation matrices. On the right, a close-up of the initial contact zone on the left is shown. The figure illustrates how using a higher number of foundation elements, combined with the evaluation of foundation properties at Gauss points, significantly enhances the model’s ability to accurately identify the initial point of contact. This is because the beam nodes are much coarser than the Gauss points of the foundation mesh. With this formulation, the foundation mesh can be refined to the desired level of accuracy for identifying discontinuities or modeling nonlinearities in the foundation, while allowing for larger elements in the beam mesh, thereby reducing the overall computational effort. In Figure 6, the foundation stiffness per unit of length and the foundation contact force per unit of length are reported. Outside the contact area, both stiffness and force are null.

**Figure 7:**
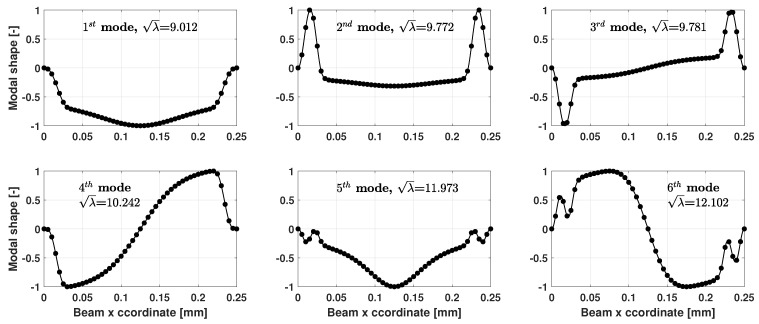
First six modal shapes of the microbeam and frequency parameters $\sqrt{\lambda }$.

Once the contact area is computed, under the hypothesis of small deformation of the microbeam, the proposed model can be utilized to compute the eigenfrequencies and the model shapes of the beam when in contact with the nonlinear elastic foundation. The first six modal shapes of the microbeam, along with the frequency parameters, are depicted in Figure 7. The modal shapes show that the vibration characteristics of the microbeam are quite different inside or outside the contact area. If larger deformations of the microbeam are present, and if the beam nonlinearities cannot be neglected, the approach described in the paper can still be applied, provided that a suitable nonlinear model of the beam elements is used.

Finally, in the presented examples, analytical expressions have been used to describe the nonlinear elastic foundation stiffness. However, since the proposed method employs Gauss integration for constructing the foundation elements, this is not a requirement. Tabular or discontinuous elastic characteristics can also be used without any modification to the described formulas. The only requirement is that the stiffness value and its derivative exist at the Gauss points.

## 6. Conclusions

This paper presents an approach for modeling microbeams on elastic foundations. The method is based on independent finite element modeling of both the beam and the elastic foundation. The two components are then coupled using a variable reduction technique, in which only the degrees of freedom at the beam nodes are retained. This allows the mesh of the elastic foundation to be refined without increasing the total number of degrees of freedom and consequently, without increasing computational time.

A nonlinear model of the elastic foundation accounting for the nonlocal effects of the microbeam was developed. The model also accounts for gaps between the foundation and the beam, and is applicable to both static and frequency analyses. Its validity was demonstrated through comparison with previously published nonlinear foundation models. Compared to existing methods, the proposed approach offers a simpler yet more flexible formulation.

An example involving a nonlinear elastic foundation with gaps illustrates the capabilities of the proposed method. In particular, it shows how using different mesh resolutions for the beam and the foundation improve the accuracy of contact area identification, while allowing larger elements to be used for the beam. Once the contact area is accurately identified, the resulting finite element model can be effectively used for frequency analysis of the system.

## Figures and Tables

**Table 1:** Comparison of the frequency parameter $\sqrt{\lambda }$ computed by the presented model and the reference model of [16] for a microbeam resting on a constant, linear, quadratic, and sinusoidal elastic foundation.

Foundation Type	Presented Model	Reference Model
Constant	4.794 5.036 5.384	4.794 5.036 5.384
Linear	3.858 4.479 4.952	3.858 4.479 4.952
Quadratic	4.218 4.735 5.124	4.218 4.735 5.124
Sinusoidal	3.977 4.524 4.986	3.977 4.524 4.986

## Data Availability

No new data were created or analyzed in this study. Data sharing is not applicable to this article.

## Appendix A

In Table A1, additional comparisons compared to those given in Section 5.1 are reported for different values of the nonlocal coefficient.

**Table A1:** A comparison of the frequency parameter $\sqrt{\lambda }$ computed by the presented model and the reference model of [16] for a microbeam resting on constant, linear, quadratic, and sinusoidal elastic foundation at different values of the nonlocal coefficient.

Nonlocal Coefficient $\frac{{\left(e_{0}a\right)}^{2}}{l}=0$		
Foundation Type	Presented Model	Reference Model
Constant	4.944 6.736 9.571	4.944 6.736 9.571
Linear	4.300 6.525 9.499	4.300 6.525 9.499
Quadratic	4.607 6.604 9.524	4.607 6.604 9.524
Sinusoidal	4.348 6.541 9.504	4.348 6.541 9.504
**Nonlocal Coefficient** $\frac{{\left(e_{0}a\right)}^{2}}{l}=1$		
**Foundation Type**	**Presented Model**	**Reference Model**
Constant	4.750 4.817 4.924	4.750 4.817 4.924
Linear	3.478 4.002 4.316	3.478 4.002 4.316
Quadratic	3.839 4.346 4.608	3.839 4.346 4.608
Sinusoidal	3.704 4.100 4.371	3.704 4.100 4.371

Note: The values of the reference model that are not explicitly reported in [16] were computed using the model described in the considered reference.
